# Facilitation of Task Performance and Removal of the Effects of Sleep Deprivation by an Ampakine (CX717) in Nonhuman Primates

**DOI:** 10.1371/journal.pbio.0030299

**Published:** 2005-08-23

**Authors:** Linda J Porrino, James B Daunais, Gary A Rogers, Robert E Hampson, Sam A Deadwyler

**Affiliations:** **1** Department of Physiology and Pharmacology, Wake Forest University Health Sciences, Winston-Salem, North Carolina, United States of America,; **2** Cortex Pharmaceuticals, Irvine, California, United States of America; University of EdinburghUnited Kingdom

## Abstract

The deleterious effects of prolonged sleep deprivation on behavior and cognition are a concern in modern society. Persons at risk for impaired performance and health-related issues resulting from prolonged sleep loss would benefit from agents capable of reducing these detrimental effects at the time they are sleep deprived. Agents capable of improving cognition by enhancing brain activity under normal circumstances may also have the potential to reduce the harmful or unwanted effects of sleep deprivation. The significant prevalence of excitatory α-amino-3-hydroxy-5-methyl-4-isoxazolepropionic acid (AMPA) glutamatergic receptors in the brain provides a basis for implementing a class of drugs that could act to alter or remove the effects of sleep deprivation. The ampakine CX717 (Cortex Pharmaceuticals), a positive allosteric modulator of AMPA receptors, was tested for its ability to enhance performance of a cognitive, delayed match-to-sample task under normal circumstances in well-trained monkeys, as well as alleviate the detrimental effects of 30–36 h of sleep deprivation. CX717 produced a dose-dependent enhancement of task performance under normal alert testing conditions. Concomitant measures of regional cerebral metabolic rates for glucose (CMR_glc_) during the task, utilizing positron emission tomography, revealed increased activity in prefrontal cortex, dorsal striatum, and medial temporal lobe (including hippocampus) that was significantly enhanced over normal alert conditions following administration of CX717. A single night of sleep deprivation produced severe impairments in performance in the same monkeys, accompanied by significant alterations in task-related CMR_glc_ in these same brain regions. However, CX717 administered to sleep-deprived monkeys produced a striking removal of the behavioral impairment and returned performance to above-normal levels even though animals were sleep deprived. Consistent with this recovery, CMR_glc_ in all but one brain region affected by sleep deprivation was also returned to the normal alert pattern by the drug. The ampakine CX717, in addition to enhancing cognitive performance under normal alert conditions, also proved effective in alleviating impairment of performance due to sleep deprivation. Therefore, the ability to activate specific brain regions under normal alert conditions and alter the deleterious effects of sleep deprivation on activity in those same regions indicate a potential role for ampakines in sustaining performance under these types of adverse conditions.

## Abstract 2

Decline in cognitive performance and changes in neural activity associated with sleep deprivation can be reversed by an AMPA receptor agonist.

## Introduction

The importance of α-amino-3-hydroxy-5-methyl-4-isoxazolepropionic acid (AMPA) receptors in learning and memory is becoming increasingly more apparent [1]. Recent studies have demonstrated that experience may directly alter GluR1 receptors in glutamatergic synaptic sites as a means by which associative learning is manifested [2], hence agents that modulate AMPA receptor function would provide a basis for enhancing these same glutamatergic synaptic processes. Ampakines are positive modulators of AMPA receptors, which bind allosterically to GluR1–GluR4 subunits and limit desensitization, thus provoking an increase in amplitude and/or duration of ionic current though the glutamate-gated channel [3–5]. Ampakines have been shown to facilitate performance in several behavioral paradigms in rodents and primates [6–9] and to enhance cognition in humans [10–12]. The ubiquitous distribution of AMPA receptor subtypes on various types of neurons in nearly every brain region [13,14] provides a feasible substrate for ampakines to enhance brain function under normal conditions as well as in more difficult or disruptive circumstances where performance may be compromised.

One circumstance that has been consistently shown to interfere with the performance of complex behavioral tasks and cognition in both humans and animal models is prolonged sleep deprivation [15–19]. There are societal as well as health-related reasons for counteracting the effects of sleep deprivation such as those encountered by individuals subjected to extended work hours or night shifts [20–23]. In this study we employed a monkey model of cognition [24] to examine whether ampakines would enhance task performance under normal alert conditions, as well as attenuate demonstrated detrimental effects of 30–36 h of sleep deprivation in the same task. We report here that the ampakine CX717 (Cortex Pharmaceuticals) produced a dose-dependent enhancement of performance of a delayed-match-to-sample (DMS) task in normal alert monkeys, and that this property extended to complete restoration of performance impaired by sleep deprivation. To gain insight into the neural basis for the CX717-induced positive alterations in performance, concomitant brain imaging of regional cerebral metabolism with positron emission tomography (PET) was performed in the same animals. The results revealed key brain areas engaged by the task, which under normal alert conditions showed increased activation by CX717. In addition, CX717 ameliorated the effects of sleep deprivation on brain metabolic changes in most of these regions and restored task-dependent activation patterns to those obtained under normal alert conditions.

## Results

### DMS Performance and Its Facilitation by CX717

#### Normal alert DMS performance

The multi-image DMS task utilized in these studies is illustrated in Figure 1 (see Materials and Methods). Performance in the task (expressed as percentage of correct trials) averaged 75% ± 0.3% over 11 monkeys tested under normal alert with vehicle injection (hereafter termed “normal vehicle”) conditions as shown in Figure 2A. The task produced a delay-dependent decrease in performance accuracy (F_[5,1759]_ = 11.42, *p* < 0.001) as well as a decrease associated with the number of images presented in the match phase of the task (F_[4,1759]_ = 19.54, *p* < 0.001). There was also a significant interaction between delay and number of images (F_[20,1759]_ = 12.11, *p* < 0.001), indicating that at longer delays, the number of images produced a more pronounced decrease in performance (difference between curves in Figure 2A). Variability across animals was minimal (F_[1,1759]_ = 1.06, not significant [NS]) and is signified by the error bars for each mean in this and all subsequent Figures. The influence of both the delay and number of image parameters was also reflected by the change in latency to make the match response that was independent from the accuracy measure (percent correct). This relationship is depicted in Figure 2B (indicated as follows: *F_[1,1176]_ > 6.65, *p* < 0.01 and **F_[1,1176]_ > 10.88, *p* < 0.001) and shows that match response latencies were influenced by number of images and length of delay (number of images, F_[4,1176]_ = 25.34, *p* < 0.001; delay, F_[5,1176]_ = 2.61, *p* < 0.02). Mean match response latency across all error trials was not significantly different from the shortest delay and fewest number of images on correct trials (F_[1,1176]_ = 1.47, NS), irrespective of trial type.

**Figure 1:**
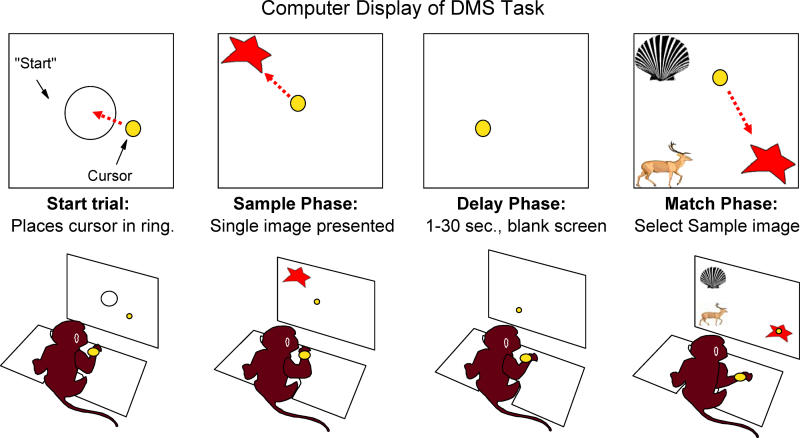
Multi-Image Visual DMS Task DMS trials consist of four phases. In the first, the start-trial phase, a circle was displayed to initiate the trial. The monkey had to move the cursor into the circle to initiate presentation of the sample image. Next, in the sample phase, a single “sample” clip art image (red star) was presented. Movement of the cursor into the sample image blanked the screen and initiated the delay phase. In this third phase, delay, the screen was blanked for a 1–30 s interval (randomly selected on each trial); the match phase was initiated automatically at the end of delay phase. In the last, match phase, the sample image was presented simultaneously with one to five other randomly chosen “nonmatch” images. Images could appear randomly at any of nine spatially distinct screen locations. The sample image always appeared at a different location in the match phase, and monkeys were required to place the cursor in the sample image for 200 ms or longer to be rewarded and terminate the trial. Movement of cursor into a nonmatch image for 200 ms or longer terminated the trial with no reward. Each clip-art image (including samples and nonmatches) was selected from a list of 5,000 clip art images and presented only once, such that all trials were unique with no repetition of images during the session. The intertrial interval was 10 s, in which the screen was blank prior to the appearance of the start-trial circle. DOI: 10.1371/journal.pbio.0030299g001

**Figure 2:**
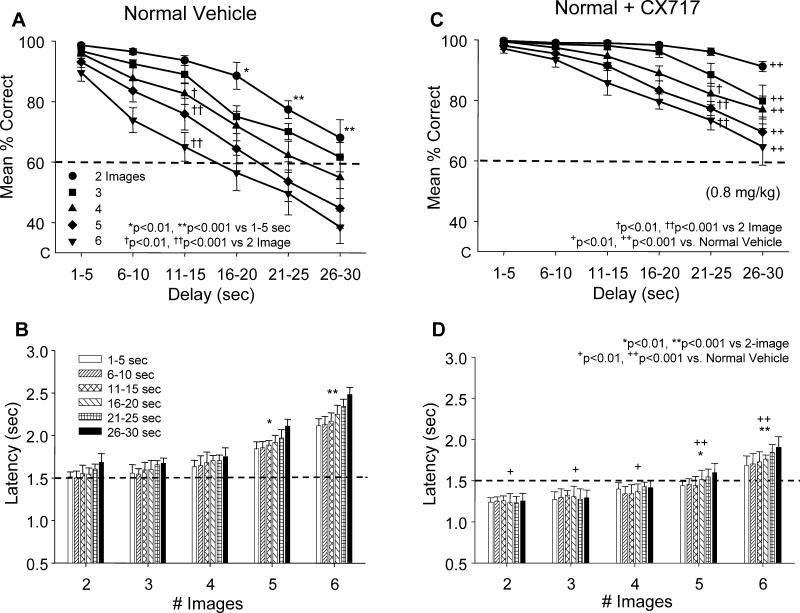
DMS Performance and Match Response Latency under Normal Vehicle and Normal + CX717 Conditions (A) Normal vehicle condition, mean (± SEM) percent correct DMS performance across 11 monkeys. Each curve reflects percent correct DMS trials sorted by number of images (two to six) in the match phase on trials with different delays (1–30 s plotted in 5-s increments). Performance decreased both as a function of delay and number of images as indicated by separation of curves across delays. “C” on y-axis indicates random or “chance” performance level in task. Asterisks indicate degree of separation from 1–5 s delay trials for significant (*p < 0.01, **p < 0.001) differences, and daggers indicate minimum degree of separation for significance (†*p* < 0.01, ††*p* < 0.001) compared to two-image trials. (B) Match phase response latencies in s (mean ± SEM) for trials shown in (A). Latencies on correct trials were sorted and plotted by number of images at specified delays. Asterisks indicate significant differences (*p < 0.01, **p < 0.001) compared to the mean of two-image trials summed across all delays (dashed line). (C) Normal + CX717 condition; mean (± SEM) percent correct performance by the same animals as in (A) following administration of CX717 (0.8 mg/kg, IV) prior to start of session. Legend is the same as in (A) for number of images and duration of delay. Performance across both parameters was maximally facilitated as indicated by comparison to dashed line arbitrarily drawn at 60%. Daggers indicate significant difference compared to two-image trials (†*p* < 0.01, ††*p* < 0.001). Plus signs indicate significant mean differences (^+^ *p* < 0.01, ^++^ *p* < 0.001) compared to the respective number of images in normal vehicle condition. (D) Match response latencies in s (mean ± SEM) for normal + CX717 condition sorted by delay and number of images as in (B). Dashed line provides a basis for comparison with minimum latency (1.5 s) in normal vehicle condition (B). Asterisks indicate significant differences (*p < 0.01, **p < 0.001) at all delays compared to two-image trials. Plus signs indicate significant differences (^+^ *p* < 0.01, ^++^ *p* < 0.001) compared to the same trial type in normal vehicle condition (B). DOI: 10.1371/journal.pbio.0030299g002

#### CX717 enhancement of normal alert DMS performance

Figure 2C shows DMS performance when CX717 (0.8 mg/kg, intravenous [IV]) was administered to normal alert animals (a condition hereafter termed “normal + CX717”). CX717 markedly improved mean overall DMS performance by 15% ± 2.5% (normal vehicle, 74.9% ± 2.9% versus normal + CX717, 90.5% ± 1.3%; F_[1,1759]_ = 17.49, *p* < 0.001) as indicated by comparison with Figure 2A (see dashed line at 60%). The effect of CX717 was so facilitative as to render mean performance on trials with delays of 10 s or less nearly perfect (mean 95% or greater, irrespective of number of images). On more difficult trials with delays of 21–30 s and five or six images, the overall mean increase of 25% in performance in the normal + CX717 condition relative to normal vehicle condition was also highly significant (mean normal vehicle, 46.7% ± 4.8% versus mean normal + CX717, 71.7% ± 2.4%; F_[1,1759]_ = 26.46, *p* < 0.001). Figure 2D shows markedly decreased match response latencies at all delays and #image trial types relative to the normal vehicle condition (overall F_[1,1176]_ = 14.70, *p* < 0.001; specific comparisons with normal vehicle indicated in Figure 2D as follows: ^+^F_[1,1176]_ > 7.77, *p* < 0.01 and ^++^F_[1,1176]_ > 12.55, *p* < 0.001), but the trend for increased latencies as a function of longer delays and increased number of images was not eliminated by CX717 (F_[20,1176]_ = 4.21, *p* < 0.01). Mean start-trial and mean sample response latencies (see Figure 1) were also significantly reduced from normal vehicle sessions (start-trial, F_[1,1176]_ = 15.23, *p* < 0.001; sample, F_[1,1176]_ = 18.46, *p* < 0.001); however, these latencies were not sensitive to trial type.

### Dose-Related Facilitation of DMS Performance by CX717

Figure 3A shows the dose-dependent facilitation of DMS performance by CX717 in nine monkeys in individual normal + CX717 sessions (dose range, 0.3–1.5 mg/kg). Daily sessions employing different doses were interleaved with vehicle sessions and showed consistency across monkeys with respect to degree of facilitation. Daily performance on vehicle sessions ranged between 70%–84% correct trials, while performance on the individual interleaved drug sessions ranged between 81%–98% correct (Figure 3A; “C”on x-axis and arrow denote drug day). The dose of 1.5 mg/kg produced a mean overall increase of 15.1% ± 0.3% (F_[1,1759]_ = 12.98, *p* < 0.001) and the 0.8 mg/kg dose a mean overall increase of 12.2% ± 0.5% (F_[1,1759]_ = 7.02, *p* < 0.01), while doses of 0.5 mg/kg or less were not as efficacious (mean increase, 6.1% ± 0.2%, F_[1,1759]_ = 3.53, NS). The onset and selectivity of the facilitative effects of CX717 on DMS performance were further demonstrated in four animals for which CX717 (0.8 mg/kg) was not administered until halfway through the session (split-session, Figure 3B). Performance improved by 12.8% ± 0.5% (F_[1,1759]_ > 13.91, *p* < 0.001) during the second versus the first half of the session. The overall means for the three different doses of CX717 across all animals tested (*n* = 9) are shown in Figure 3C. Response latencies for start-trial, sample, and match were also decreased in a dose-dependent manner (all F_[2,1176]_ > 10.30, *p* < 0.001).

**Figure 3:**
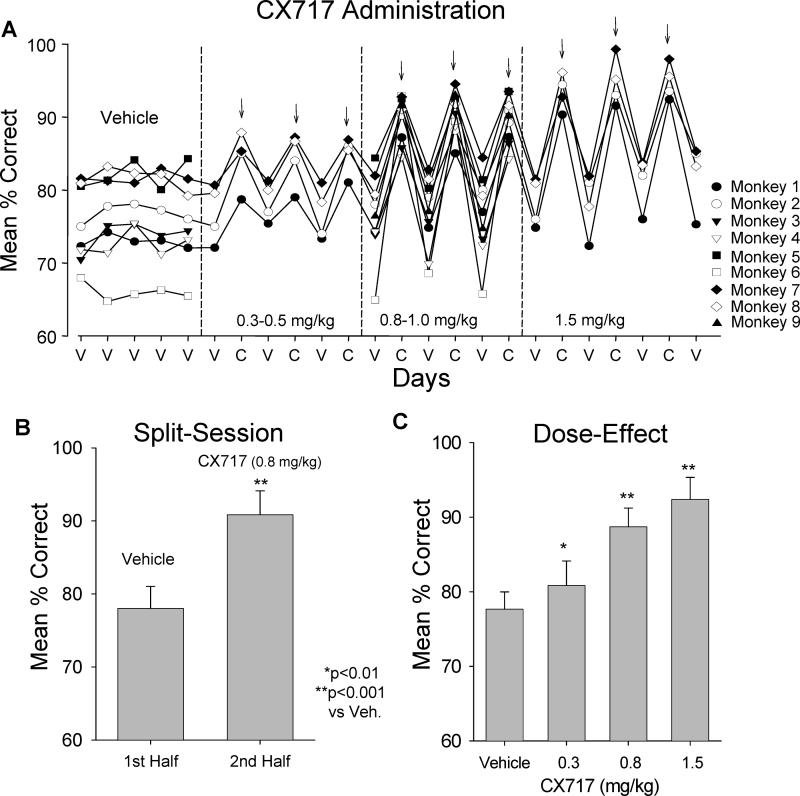
Effects of Different Doses of CX717 on DMS Performance in Normal + CX717 Condition (A) CX717 is shown administered on consecutive sessions for nine monkeys over three dose ranges of CX717 (0.3–0.5 mg/kg, 0.8–1.0 mg/kg, and 1.5 mg/kg, IV). Each CX717 session (C, arrows) was interspersed with a single normal vehicle (V) session. Curves show mean (± SEM) percent correct performance over the entire session from each different monkey, as indicated by separate symbols. Arrows indicate CX717 sessions (also denoted by “C” on axis). Note escalating doses (0.3, 0.8, and 1.5 mg/kg) shown were in four of the nine monkeys. (B) Performance on split sessions (*n* = 4 monkeys) in which vehicle was administered at the start of the DMS session, and midway through same session CX717 (0.8 mg/kg, IV) was administered via remote pump without interruption. Mean (± SEM) percent correct performance over at least 50 trials was calculated separately for the first half (vehicle) and second half (CX717, 0.8 mg/kg) of the same session. Asterisks indicate a significant (** *p* < 0.001) increase in the second half of the session relative to the first (vehicle). (C) Overall mean dose-effect relationship of CX717 on normal alert DMS performance across monkeys (*n* = 9) in sessions in which each of the three doses (0.3, 0.8, and 1.5 mg/kg, IV) was administered. DMS sessions in which the three doses were received in an escalating order were compared with sessions in which the same doses were administered in a randomized order (F_[1,50]_ = 2.31, *p* = 13.5, NS). Asterisks indicate significant (*p < 0.01, **p < 0.001) difference relative to vehicle sessions. DOI: 10.1371/journal.pbio.0030299g003

### Effects of Sleep Deprivation on DMS Performance and Restoration by CX717

#### Effects of sleep deprivation on DMS performance

Figure 4A shows that 30–36 h of sleep deprivation (with vehicle; hereafter termed “sleep deprivation”) significantly reduced overall DMS performance from the normal vehicle condition (mean performance with sleep deprivation, 62.7% ± 2.5%, F_[1,1759]_ = 15.42, *p* < 0.001). The effects of sleep deprivation on performance were not differential with respect to delay, since there was a major deficit in performance (10.3% overall decrease) on long-delay trials with increased number of images (21–30 s with five and six images: mean normal vehicle, 46.7% ± 3.0% versus mean sleep deprivation, 36.4% ± 1.9%; F_[1,1759]_ = 8.62, *p* < 0.01) and a marked 9.1% decrease on trials with the shortest (1–10 s) delays and the fewest (two and three) number of images (mean normal vehicle, 95.8% ± 1.1% versus mean sleep deprivation, 86.7% ± 3.0%; F_[1,1759]_ = 8.33, *p* < 0.01; compare with dotted line in Figure 2A). This was also reflected by the significant overall proportionate change (118% ± 6%) in the match response latency distribution (Figure 4B) with no differential effect on any one trial type. Although not significant for two images, sleep deprivation increased latencies systematically at three to six images, showing a similar interaction with delay as in the normal vehicle condition (see Figure 2B) but to a much greater extent (overall, F_[1,1176]_ = 17.45, *p* < 0.001; comparison with normal vehicle condition indicated on Figure 2B as follows: ^+^F_[1,1176]_ > 7.17, *p* < 0.01; ^++^F_[1,1176]_ > 10.28, *p* < 0.001). Significant increases in mean latency were also exhibited in the start-trial and sample response latencies (see Figure 1) of the task (start-trial, F_[1,1176]_ = 11.46, *p* < 0.001; sample, F_[1,1176]_ = 13.91, *p* < 0.001); however, they did not increase systematically with trial type.

**Figure 4:**
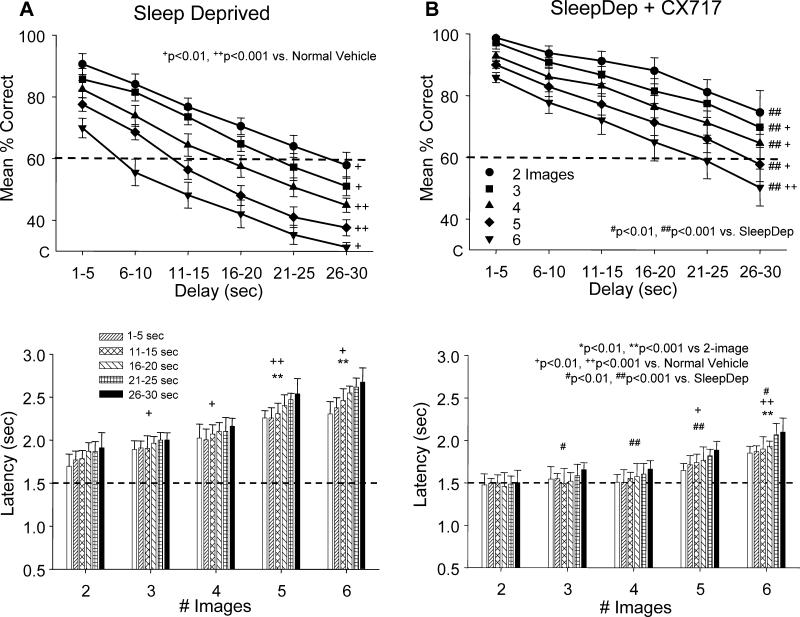
Effects of Sleep Deprivation and Sleep Deprivation + CX717 (A) Sleep deprivation condition disrupts DMS performance. Mean percent correct performance across animals for sessions following 30–36 h of sleep deprivation (nine of 11 monkeys in Figure 2A). Plus signs indicate significant mean differences (^+^ *p* < 0.01, ^++^ *p* < 0.001) compared to the respective number of images curve in the normal vehicle condition; dashed line at 60% allows comparison with Figure 2A. (B) Match response latencies in s (mean ± SEM) sorted for different trial types as in Figure 2B. Asterisks indicate significant difference (*p < 0.01, **p < 0.001) at all delays compared to two-image trials. Plus signs indicate significant differences (^+^ *p* < 0.01, ^++^ *p* < 0.001) compared to same trial types, and dashed line allows comparison with minimum latencies in normal vehicle condition (Figure 2B)**.** (C) Sleep deprivation + CX717 condition. Effects of administration of CX717 (0.8 mg/kg, IV) 10 min prior to DMS session following 30–36 h of sleep deprivation. Mean (± SEM) percent correct DMS trials for same delay (1–30 sec, in 5-s increments) and #image (two to six) DMS conditions generated by same monkeys tested in (A). Plus signs indicate significant mean differences (^+^ *p* < 0.01, ^++^ *p* < 0.001) compared to the respective number of images curve in the normal vehicle condition (Figure 2A); pound signs (^#^ *p* < 0.01,^##^ *p* < 0.001) indicate significant differences compared to the same trial types in sleep deprivation condition. Dashed reference line allows comparison with sleep deprivation (A) and normal vehicle conditions (Figure 2A). (D) Match response latencies for same sleep deprivation + CX717 condition shown in (C), sorted by delay and number of images and labeled as in Figure 2B. Pound signs (^#^ *p* < 0.01, ^##^ *p* < 0.001) indicate significant differences compared to the same trial types in sleep deprivation condition (A). DOI: 10.1371/journal.pbio.0030299g004

#### Effects of CX717 on DMS performance in sleep-deprived animals

When CX717 (0.8 mg/kg IV) was administered 10 min prior to testing animals that were 30–36 h sleep deprived (hereafter termed “sleep deprivation + CX717”), the detrimental effects on DMS performance were eliminated. Figure 4C shows that DMS performance in sleep-deprived animals was markedly improved (mean sleep deprivation + CX717, 84.5% ± 2.0% versus mean sleep deprivation, 62.7% ± 2.5%; F_[1,1759]_ = 21.71, *p* < 0.001) and in fact, was significantly elevated relative to the normal vehicle condition (compare with dotted line in Figure 2A) as indicated by a overall mean increase of 9.6% ± 2.5% (F_[1,1759]_ = 9.43, *p* < 0.001). This reversal by CX717 was characterized by a marked shift to shorter match response latencies relative to the sleep deprivation condition (indicated on Figure 4D as follows: ^#^F_[1,1176]_ > 8.50, *p* < 0.01, ^##^F_[1,1176]_ > 11.14, *p* < 0.001) and by smaller differences in match response latency between trial types within the sleep deprivation + CX717 condition (F_[1,1759]_ = 7.86, *p* < 0.01). In addition, match response latencies were significantly shorter (indicated on Figure 4D as follows: ^+^F_[1,1176]_ > 6.78, *p* < 0.01; ^++^F_[1,1176]_ > 10.74, *p* < 0.001) than in the normal vehicle condition (dotted line in Figure 4D). The changes in performance produced in sleep-deprived monkeys receiving CX717 were also reflected as decreased response latencies in the other phases of the task (start-trial, F_[1,1176]_ = 15.58, *p* < 0.001; sample, F_[1,1176]_ = 12.81, *p* < 0.001; match, F_[1,1176]_ = 17.25, *p* < 0.001 versus sleep deprivation), but again, not associated with trial type.

### Effects of Sleep Deprivation and CX717: Delay and Number of Images

Figure 5 summarizes the effects of all four testing conditions collapsed across the two main DMS task parameters of delay (Figure 5A) and number of images (Figure 5B). It is clear that both parameters controlled performance in all four test conditions in the same manner, with performance decreasing as a function of both duration of delay (overall effect of delay, F_[5,1759]_ = 10.44, *p* < 0.001; Figure 5A) and number of images (overall effect of number of images, F_[4,1759]_ = 6.49, *p* < 0.001; Figure 5B). Administration of CX717 (0.8 mg/kg IV, squares) markedly improved performance relative to normal vehicle sessions with respect to both parameters (delay, F_[5,1759]_ > 12.21, *p* < 0.001; number of images, F_[4,1759]_ > 15.22, *p* < 0.001; Figure 5, squares), while sleep deprivation produced significant impairment with respect to these two task parameters (Figure 5, triangles). Administration of CX717 (0.8 mg/kg IV) not only reversed performance deficits in the sleep deprivation condition, but also increased levels relative to the normal vehicle condition across the two parameters commencing at delays of more than 10 s (compare diamonds and circles in Figure 5 at points indicated by asterisks; delay, F_[5,1759]_ = 9.64, *p* < 0.001; number of images, F_[4,1759]_ = 10.20, *p* < 0.001).

**Figure 5:**
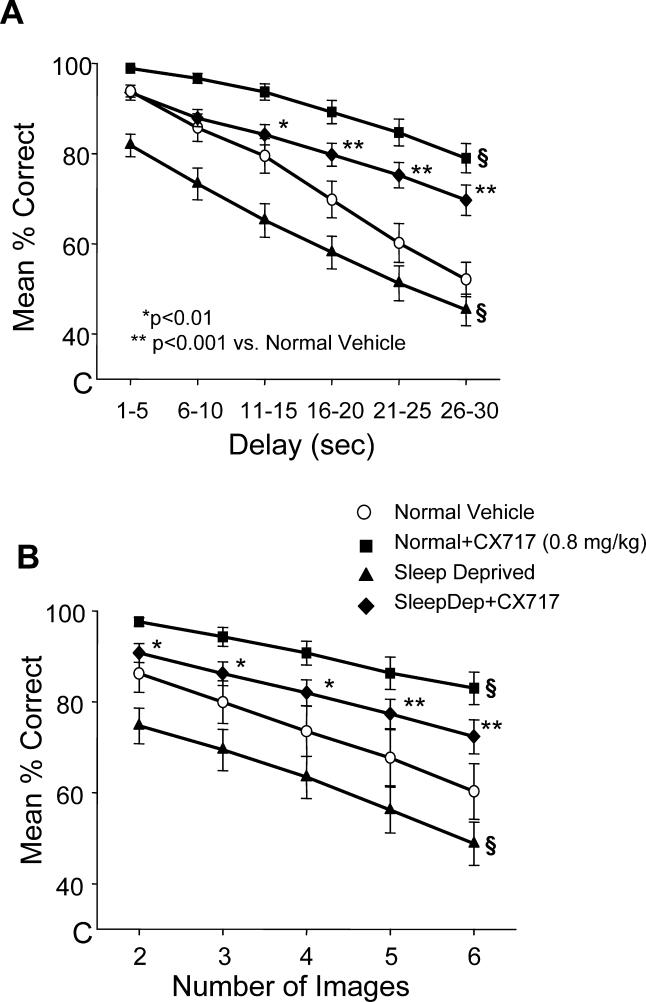
Separation of Effects of Sleep Deprivation + CX717 on DMS Performance Parameters, Delay Versus Number of Images (A) Mean (± SEM) percent correct performance across animals on DMS trials sorted by delay (1–30 sec, in 5 s increments) and summed over two to six image trials for normal vehicle, normal + CX717, sleep deprivation, and sleep deprivation + CX717 conditions (separate curves). Data are the same as shown in Figures 2 and 4. (B) Mean (± SEM) percent correct performance for DMS trials partitioned by number of images and summed over 1–30 s delays for the same four treatment conditions shown in (A). Asterisks indicate separation limits for significant differences (*p < 0.01, **p < 0.001) of CX717 and sleep deprivation means compared to the normal vehicle mean; **§** symbol indicates that all points on the indicated curve differ significantly from all points on normal vehicle curve. DOI: 10.1371/journal.pbio.0030299g005

### Electroencephalogram Correlates of Sleep and Effects of CX717

Surface electroencephalogram (EEG) was recorded during DMS testing in four animals utilizing a configuration of electrodes inserted through a craniotomy access cylinder positioned over the parietal cortex. Power spectral density (PSD) analyses were calculated from the EEG sequentially in 4.0s epochs and averaged over 10-min segments for statistical comparisons. Mean PSD graphs shown in Figure 6 (upper graphs) reveal marked changes in the EEG characteristic of sleep deprivation relative to the normal vehicle condition including (1) a specific increase in power of the “delta” (0.5–5 Hz) band reported to be associated with slow-wave sleep [25–27], and (2) peaks in the “beta” band (18–25 Hz) reported to increase during REM sleep [28,29]. Across all four animals, these two changes were significantly correlated with decreased accuracy of performance in the same testing sessions (percent correct, *r^2^* = 0.72, F_[1,128]_ = 9.94, *p* < 0.01; Figure 4A). Individual frequency shifts in the EEG, as indicated by the lower traces in Figure 6, were assessed by PSD analyses in 4.0 s epochs across the entire session and showed marked elevations in the number of transient incidences in which power in the delta and beta bands increased in sleep-deprived animals. Such changes have been reported to reflect occurrences of “microsleep” episodes [30,31]. In the sleep deprivation + CX717 condition there was a distinct shift toward the normal vehicle profile of EEG and PSD traces (Figure 6, right) characterized by a marked reduction of power (F_[2,61]_ = 6.17, *p* < 0.01) in the beta band and slower delta frequencies (Figure 6, right).

**Figure 6:**
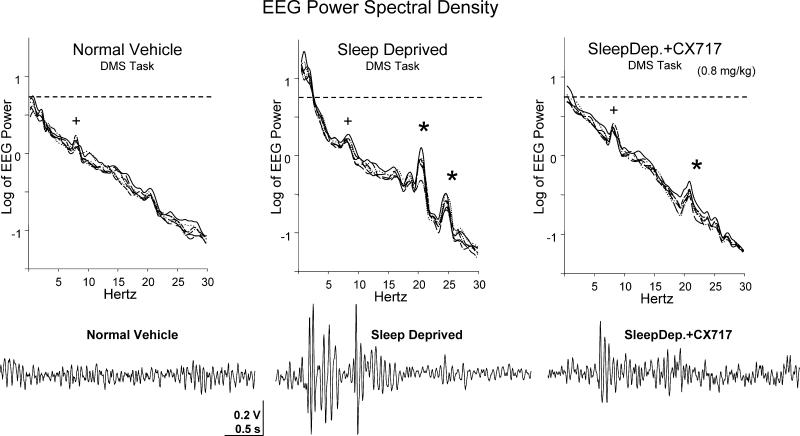
EEG and PSD Measures in Sleep-Deprived Monkeys Measures superimposed (*n* = 4) are average PSD (upper graphs) and individual sample EEGs (lower tracings), recorded from electrodes placed over the parietal cortex, shown for three different treatment conditions (left, normal vehicle; middle, sleep deprivation; and right, sleep deprivation + CX717). In the graphs (upper) are shown PSDs for EEGs recorded from animals performing the DMS task. Sleep deprivation condition (middle) shows increased power of high-amplitude, slow-wave delta band (0.5–5 Hz, above dotted line) and two prominent peaks in the beta band (18–25 Hz) at 20 and 25 Hz (asterisks) relative to the normal vehicle condition (left). Reduction of sleep deprivation features of PSD in same animals following administration of CX717 (0.8 mg/kg) is indicated at right by a decrease in PSD delta band (0.5–5 Hz, dotted line) and reduction in power of peaks (asterisks) in the beta band (18–25 Hz). PSDs were calculated as log of root-mean-square power in 10-min segments of EEG recordings, using frequency increments of 0.25 Hz, plotted separately for four monkeys in each condition (individual traces). In the tracings (lower) are shown EEG segments (4.0 s) recorded from one of five electrode locations on the surface of the parietal cortex to demonstrate different EEG frequencies present in each condition. Traces correspond to the respective DMS testing sessions shown in the PSD plots above. DOI: 10.1371/journal.pbio.0030299g006

### Rates of Local Cerebral Metabolism during Task Performance

Statistical parametric maps using SPM99 software were generated for comparisons of relative cerebral metabolic rates for glucose (CMR_glc_) across nine monkeys (Figure 7). When compared to the “neutral” baseline scan condition consisting of a video viewed in lighting and audio conditions identical to task conditions with no arm movements required, relative CMR_glc_ during the DMS task was increased in the premotor, motor, and somatosensory cortices of the left hemisphere as shown in Figure 7A, 7B, and 7D (horizontal and coronal views). These asymmetric activations reflect the use of the contralateral (right) arm and hand to perform the DMS task versus the baseline condition, and as such provide a validation of CMR_glc_ as an accurate indicator of functional neuronal activity expressed within specific brain regions during the task. Further verification was provided by increased bilateral activation detected in the cortical frontal eye fields (Figure 7A and 7B) relative to baseline, reflecting extensive eye movements during visual scanning required by the task (see Figure 1).

**Figure 7:**
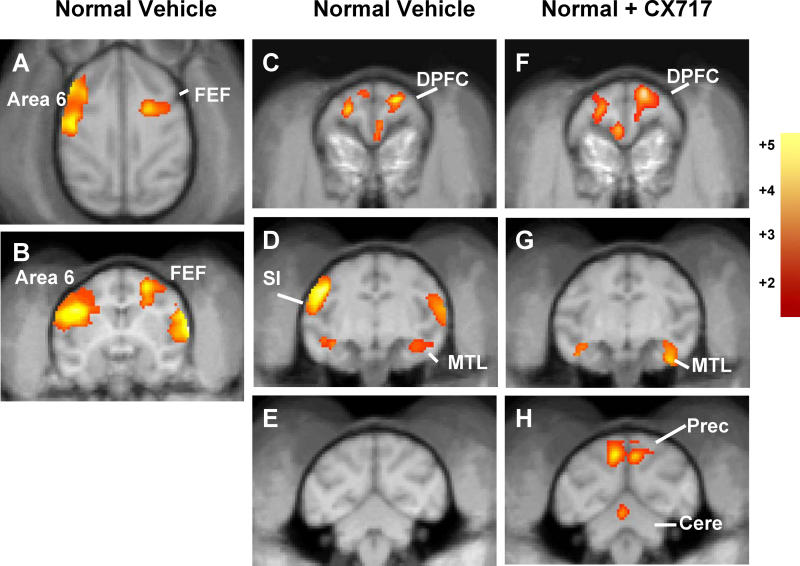
SPM Maps of Brain Regions Showing Changes in Regional CMR_glc_ during Performance of DMS Task for Normal Vehicle and Normal + CX717 Conditions (A–E) Maps are shown for the comparison of CMR_glc_ during DMS performance in the normal vehicle to the baseline, no-task condition. CMR_glc_ was significantly increased during normal vehicle condition compared to baseline, no-task condition. (F–H) Maps are shown for the comparison of CMR_glc_ during DMS performance in the normal + CX717 condition to normal vehicle condition. CMR_glc_ was increased following the administration of CX717 (0.8 mg/kg, IV) as compared to normal vehicle condition (C–E). Regional changes are displayed on both horizontal section (A) and coronal sections (B–H) of MR images of rhesus monkey brain at the level of the motor and premotor cortex (A and B), DPFC (C and F), MTL (D and G), and parietal cortex (E and H). Statistical parametric maps were generated using SPM99 software. Colors indicate the location of clusters with a height (magnitude) threshold of *p* < 0.05 and spatial extent greater than 50 voxels. Color bar indicates *t* values for the comparisons (red, *t* = 2.0, *p* < 0.05; yellow, *t* = 5.0, *p* < 0.001). Note: left and right half of hemisphere are shown on left and right of images respectively. Area 6, premotor cortex; Cere, cerebellum; FEF, frontal eye fields; Prec, precuneus; S1, primary somatosensory cortex. DOI: 10.1371/journal.pbio.0030299g007

#### CMR_glc_ during DMS task performance

Planned comparisons of relative CMR_glc_ in each of the four test conditions were made in five regions of interest (ROIs): the bilateral dorsal prefrontal cortex (DPFC), dorsal striatum (DStr), and medial temporal lobe (MTL), as well as the thalamus and precuneate cortex. Performance of the DMS task, when compared to the baseline condition, significantly increased CMR_glc_ bilaterally in DPFC (left, *t*_diff_ [paired comparisons hereafter referred to as *t*] = 3.65, *p* < 0.012; right, *t* = 3.75, *p* < 0.007) as can be seen in Figure 7C and within the MTL (Figure 7D), which included the hippocampus and surrounding medial temporal cortex (left hemisphere, *t* = 2.68, *p* < 0.02; right, *t* = 3.86, *p* < 0.02). Significant activation was also seen in the left DStr (*t* = 2.77, *p* < 0.02). No significant changes were observed in the thalamus or precuneate portion of the parietal cortex (Figure 7E).

#### Effects of CX717 on CMR_glc_

CMR_glc_ in the normal + CX717 (0.8 mg/kg, IV) condition was significantly elevated in DPFC (Figure 7F; left, *t* = 2.56, *p* < 0.03; right, *t* = 2.97, *p* < 0.02) and in MTL (Figure 7G; left, *t* = 3.06, *p* < 0.02; right, *t* = 3.67, *p* < 0.01) relative to the normal vehicle condition. The most prominent effect, however, was seen in the precuneus, where CMR_glc_ (which was not significantly altered by the task when compared to the baseline condition [Figure 7H]) was markedly increased by CX717 compared to the normal vehicle condition (*t* = 4.10; *p* < 0.003). There were no further increases in CMR_glc_ in the DStr or thalamus in the normal + CX717 condition.

#### Effects of sleep deprivation on CMR_glc_

Sleep deprivation produced both increases and decreases in relative CMR_glc_ (Figure 8A–8C) throughout the ROIs activated in either the normal vehicle and/or normal + CX717 conditions. Comparing the sleep deprivation condition to the DMS task condition revealed marked increases in CMR_glc_ in the MTL (Figure 8C; left, *t* = 4.36, *p* < 0.003; right, *t* = 4.09, *p* < 0.006), as well as within the precuneus (*t* = 4.06, *p* < 0.006). In contrast, Figure 8A shows that CMR_glc_ was significantly decreased bilaterally in the DPFC (left, *t* = 3.33, *p* < 0.01; right, *t* = 3.72, *p* < 0.009), and in the left DStr (Figure 8B; *t* = 3.28, *p* < 0.02). The thalamus, which was not changed from the no-task baseline in either the normal vehicle or normal + CX717 conditions, showed a very large decrease in CMR_glc_ (*t* = 3.97, *p* < 0.007) during performance of the task in the sleep deprivation condition.

**Figure 8:**
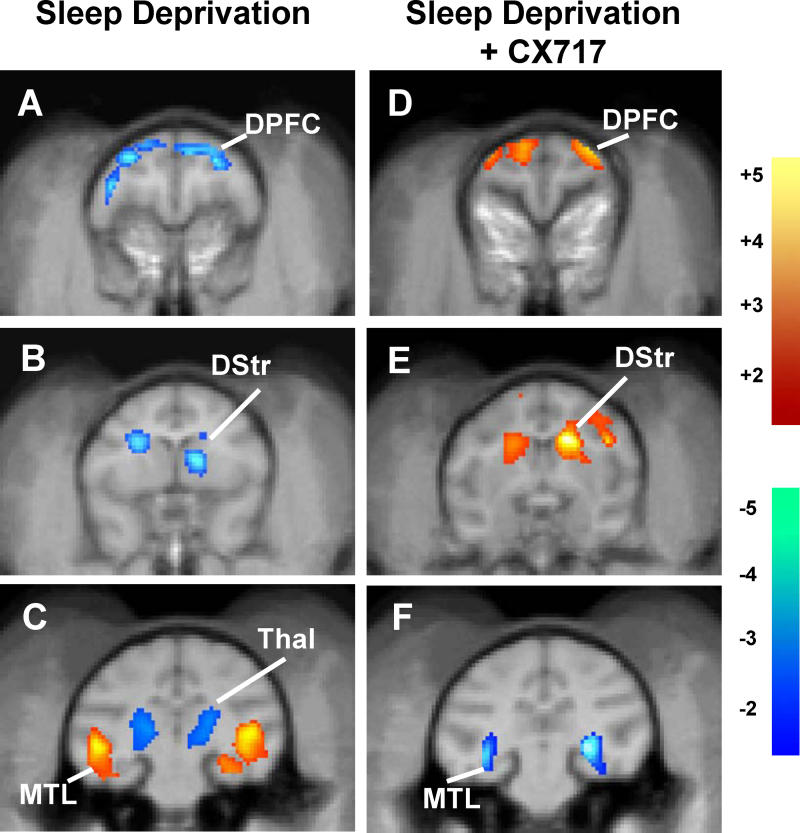
SPM Maps of Brain Regions Showing Changes in Regional CMR_glc_ During Performance of DMS Task for Sleep Deprivation and Sleep Deprivation + CX717 Conditions (A–C) Maps are shown for the comparison of CMR_glc_ during DMS performance in sleep deprivation to normal vehicle conditions (see Figure 7C–7E). Decreases (blue) are shown for DPFC (A), DStr (B), and thalamus (Thal; C). Increases in CMR_glc_ (orange and yellow) are shown in C for MTL. (D–F) Maps shown for comparison of CMR_glc_ during DMS performance in sleep deprivation + CX717 (0.8 mg/kg, IV) to the sleep deprivation condition. Increases in CMR_glc_ were observed in DPFC (D) and DStr (E), whereas decreases were observed in MTL (F). Absence of labeled voxels for thalamus (Thal) in (F) indicates no change from sleep deprivation (C above) versus sleep deprivation + CX717 conditions. Regional changes are displayed on coronal sections of MR images of rhesus monkey brain at the level of the DPFC (A,D), DStr (B,E) and MTL (C,F). Statistical parametric maps were generated using SPM99 software. Colors indicate the location of clusters with a height threshold of *p* < 0.05 and spatial extent greater than 50 voxels. Color bars indicate *t* values (2–5; corresponding to *p* < 0.05 to *p* < 0.001, respectively) for significance of increase (red-yellow) or decrease (blue-green) in comparisons. Left and right hemispheres are on the left and right of images respectively. DOI: 10.1371/journal.pbio.0030299g008

#### Effects of sleep deprivation and CX717 on CMR_glc_

The administration of CX717 (0.8 mg/kg IV) to sleep-deprived monkeys during the DMS task produced significant changes in CMR_glc_ (Figure 8D–8F) in all but one of the brain regions affected by sleep deprivation. CMR_glc_ in the DPFC (Figure 8D) was elevated bilaterally in the sleep deprivation + CX717 compared to the sleep deprivation condition (left, *t* = 3.87, *p* < 0.003; right, *t* = 3.43, *p* < 0.007). Similar increases were found bilaterally in the DStr (Figure 8E; left, *t* = 6.24, *p* < 0.001; right, *t* = 6.34, *p* < 0.004). Thus, CMR_glc_ in the DPFC and DStr, although decreased in the sleep deprivation condition, showed an increase under similar sleep-deprived conditions following CX717 administration. In contrast, significant decreases in CMR_glc_ were observed in the MTL (Figure 8F; left, *t* = 4.34, *p* < 0.007; right, *t* = 7.85, *p* < 0.001) and precuneus (*t* = 4.85; *p* < 0.002) in the sleep deprivation + CX717 condition, which constituted a directional change from the elevated level in the sleep deprivation condition. Unexpectedly, CX717 did not have a significant effect in the thalamus, where CMR_glc_ remained severely depressed as in the sleep-deprivation condition.

To assess whether the depressed CMR_glc_ in the DPFC and DStr and the increased CMR_glc_ in the MTL and precuneate regions produced by CX717 administration in sleep-deprived animals was, in effect, a shift toward normal vehicle levels, the sleep deprivation + CX717 condition and normal vehicle conditions were compared directly. This comparison showed no significant differences in CMR_glc_ in the DPFC, DStr, or MTL. In other words, there were no voxels within these ROIs that met the cluster threshold of *p* < 0.05, indicating a lack of difference between the two conditions for these regions. However, in this same comparison, CMR_glc_ in the thalamus remained significantly decreased (*t* = 5.32, *p* < 0.001).

### Multivariate Analyses of CX717 and Sleep Deprivation Effects

While the above analyses of behavioral performance (see Figures 2–5) and measures of CMR_glc_ (see Figures 7 and 8) demonstrated overall mean effects of CX717 and/or sleep deprivation summed across animals, a multivariate canonical discriminant analysis (CDA [32]) demonstrated conclusively that these measures actually covaried within individual animals across the four test conditions. The CDA identified and extracted orthogonal sources of variance between animals and treatment conditions that derived from six different measures of DMS task performance and CMR_glc_ values from six different brain regions in the same animals (see Materials and Methods). The extracted discriminant functions (DFs) are shown in Figure 9A as mean DF scores averaged across animals (mean ± standard error of the mean [SEM]). Each of the three DFs is plotted relative to its mean value in the normal vehicle condition (Figure 9A; “0” line in graph) for each of the other three test conditions (normal + CX717, sleep deprivation, and sleep deprivation + CX717).

**Figure 9:**
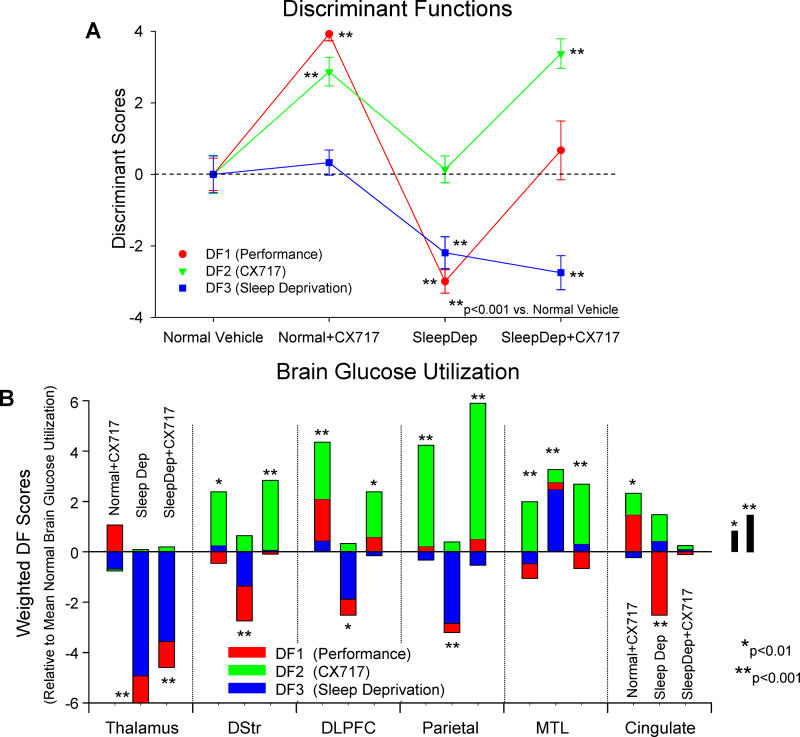
Multivariate CDA of DMS Performance and Brain CMR_glc_ Activity (A) Discriminant functions (DFs 1, 2, and 3) were extracted via CDA from covariance matrices for 12 dependent variables of DMS performance and CMR_glc_ for nine monkeys across the four treatment conditions (see Materials and Methods). Discriminant scores for each DF (different colored curves) were computed for each animal and treatment condition. Scores were rotated via the CDA for orthogonality, and then standardized with mean of zero, standard deviation ± 1.0, relative to the normal vehicle condition for comparison to the other three test conditions and plotted as means (± SEM) across all animals. Positive mean scores for DF1 indicate increased accuracy (i.e., percent correct trials) and decreased latency in DMS performance, while negative scores indicate decreased accuracy and increased latency. Note that scores for DF2 increased when DF1 increased. Scores for DF3 show only decreases associated with both sleep deprivation conditions. Asterisks indicate significant (*p < 0.01, **p < 0.001) difference compared to normal vehicle score for the respective DF. (B) A component of the CDA analysis extracted sources of variance associated with changes in absolute CMR_glc_ (see Materials and Methods) across the six ROIs described in Figures 7 and 8. The length of each bar reflects the total change in CMR_glc_ measured in that structure (i.e., the ROI). Positive scores indicate relative increases in CMR_glc_ within a brain region with respect to their levels in the normal vehicle condition (“0”). Negative scores indicate relative decreases in CMR_glc_ within a brain region. Each colored segment of the bar indicates the proportion of total CMR_glc_ variance contributed by each of the respective DFs (A) for a given treatment condition: DF1 (orange), DF2 (green), DF3 (blue). Asterisks indicate conditions with significant (*p < 0.01, **p < 0.001) differences in CMR_glc_ compared to normal vehicle. Height of small black bars at right indicate significance levels for colored segments of bars. DOI: 10.1371/journal.pbio.0030299g009

The three discriminant functions shown in Figure 9A (DFs 1, 2, and 3) accounted for 94% of total variance in the covariance matrix. The largest, DF1 (54% of total variance, F_[11,612]_ = 32.40, *p* < 0.001, relative to residual variance, red curve), was maximally positive when performance was significantly improved in the normal + CX717 condition, maximally negative when performance was severely impaired in the sleep deprivation condition, and returned to positive in the sleep deprivation + CX717 condition. The second largest source of variance, DF2 (24% of total variance, F_[11,612]_ = 13.79, *p* < 0.001, green curve), was significantly positive (relative to its value in the normal vehicle condition) whenever CX717 was administered independent of whether animals were alert or sleep deprived (i.e., normal + CX717, sleep deprivation + CX717). The third significant source of variance, DF3 (14% of total variance, F_[11,612]_ = 11.45, *p* < 0.001, blue curve), differed from the normal vehicle condition during the two sleep deprivation conditions, indicated by negative scores, irrespective of whether CX717 was administered.

A subset of the CDA covariance matrix examined CMR_glc_ measures for each of six selected brain regions (see Materials and Methods) with respect to the same three DFs shown in Figure 9A. Each bar in Figure 9B shows the mean (± SEM) discriminant score for increases (positive) and decreases (negative) in CMR_glc_ in each of the three test conditions relative to the normal vehicle condition. The colored segments of each bar reflect the proportionate degree of variance in CMR_glc_ level associated with the three DFs shown in Figure 9A within a given region (same color code). It is clear from Figure 9B that DF2 (CX717, green segments) and DF3 (sleep deprivation, blue segments) had large influences on regional CMR_glc_ and tended to vary inversely across the three test conditions. CMR_glc_ values in MTL, parietal cortex (precuneate), DStr, and DPFC were differentially influenced by DF2 and DF3 in relation to their values in the normal vehicle condition (“0” in Figure 9B). The large extracted variances associated with these regions resulted from (1) changes produced by sleep deprivation (see Figure 7E) and (2) the corresponding reversal of those effects in the sleep deprivation + CX717 condition (see Figure 8). Consistent with the above findings, the thalamus was influenced exclusively by DF3, the factor associated with sleep deprivation (blue segments in Figure 9B), showing intense reductions in CMR_glc_ levels in both sleep deprivation test conditions regardless of the presence of CX717.

## Discussion

The AMPA receptor modulator CX717, given to monkeys engaged in cognitive processing and short-term memory, improved performance in all aspects of the DMS task (see Figures 2 and 5). This enhanced performance was evidenced by significant increases in accuracy at all delays and number of images (see Figure 5) and was paralleled by reductions in latency to select the correct image during the match phase of the task (see Figure 2). In addition to the effects on normal alert performance, the present findings also demonstrated that CX717 removed severe performance deficits produced by subjecting the animals to prolonged periods of sleep deprivation (see Figure 4). In conjunction with the effects on behavioral performance, CX717 also affected CMR_glc_ in brain regions engaged while performing the task under normal and/or sleep deprived conditions (see Figures 7 and 8). The results show a strong functional relationship between the neurobiological and behavioral effects of the drug and provide insight into how performance in this cognitive task is facilitated by ampakines.

Reports from this and other laboratories have demonstrated enhanced performance by other AMPA receptor modulators under normal alert conditions [6–9], one of which employed a task with monkeys similar to the one utilized here [9]. The current findings extend the basis for the facilitative actions of ampakines to the parallel yet equally sensitive measure, match response latency (see Figures 2B, 2D, 4B, and 4D). The significant modulation of match response latencies by task parameters in the same manner as performance accuracy (percent correct trials) suggests that CX717 also facilitated attentional processes [33] related to speed of responding on successful trials (Figure 7A and 7B), even if the animals were sleep deprived (see Figure 4D). Since this measure was markedly facilitated by CX717 under normal alert conditions (see Figure 2), it likely reflects an increase in accuracy as well as speed of detection of the correct match target.

The marked influence of sleep deprivation on performance (see Figures 4 and 5) was paralleled by significant alterations in CMR_glc_ in DPFC, DStr, precuneate cortex, and MTL (Figure 8), demonstrating the contribution of a sleep-related factor to brain regions engaged by the DMS task (DF3 in Figure 9). It is also evident that both behavioral and brain metabolic processes sensitive to sleep deprivation were affected by administration of CX717 (see Figures 8 and 9). Although it is possible that the positive effect of CX717 in sleep-deprived animals was due to an increase in arousal, this explanation is unlikely because of the potent dose-dependent action of the drug in normal animals (see Figure 2B, Figure 5). The performance levels of animals in the sleep deprivation condition were reduced significantly compared to normal vehicle sessions; however, a close examination of latencies (see Figures 4 and 5) shows that animals continued to respond in the match phase in a rapid manner (2.5–3.0 sec). The assessment of EEG parameters, although not designed to provide a thorough analysis of sleep architecture, suggests that animals were experiencing microsleep episodes during the sleep deprivation testing condition [30,31]. However, these instances did not significantly alter the mean number of trials completed during sessions that were the same duration as normal vehicle tests, nor was the latency to respond to events during the trial elevated more than 1–2 s above normal vehicle sessions (see Figures 4 and 9). While preliminary analyses showed that administration of CX717 reduced the incidences of delta and beta band occurrences in sleep-deprived animals (see Figure 6), more rigorous assessments of EEG-related changes in the sleep states of the animals will be required to reveal the exact means by which this reversal occurred.

The MTL (including hippocampus) and DPFC have been extensively implicated in both working and short-term memory in monkeys and humans [34–37]. The differential changes in CMR_glc_ in these regions make them strong candidates for the functional substrates of the type(s) of memory process required to successfully perform this DMS task [24,38]. Further evidence suggests that the activation of DPFC and MTL reflected utilization of generalized encoding principles shown also in humans [39]. Although there also exist differences in humans with respect to tasks that are susceptible to sleep deprivation [40], the fact that performance of this task was severely disrupted, and that both the DPFC and MTL were affected, are consistent with studies in humans showing that repetitive working memory tasks that require a high degree of vigilance are affected by sleep deprivation in a similar manner [41]. In addition, Benca et al [26] showed that 24-h sleep architecture was drastically altered in monkeys sustaining lesions of the amygdala, an area possibly contributing to the changes in CMR_glc_ in the MTL in the current findings.

The detrimental effects on DMS performance of 30–36 h of sleep deprivation in monkeys reported here (see Figures 4A and 5) are difficult to compare directly with different durations of sleep deprivation in humans [16,42]. Given the severity and magnitude of the deficit, it is possible that this degree of sleep deprivation in monkeys has more of an effect than in humans more adapted to a 30–36 h (a single night) sleep deprivation period. The current findings are consistent with some reports in humans that show extensive sleep-deprivation-produced deficits in prefrontal-cortex-sensitive cognitive tasks [15,27,42]. However, other reports suggest that complete resolution of these issues will require confirmation that the DMS task employed here is similar to the verbal and/or arithmetic tests used to dissociate different brain regions and circuits in sleep-deprived humans [17,40].

Because of the differential actions in DLPC and DStr versus MTL, it is possible that CX717 influenced the attempts of selective neural circuits to restore total brain activation to a pattern exhibited under normal alert conditions. This would seem logical if additional mechanisms were activated under sleep deprivation conditions to compensate for decreased performance of the task [15,18,40,43]. Reciprocal changes in DPFC and MTL in the sleep deprivation versus sleep deprivation + CX717 condition may reflect switching between different brain activation patterns as indicated in Figure 9B. In this regard, the fact that the drug-related regional CMR_glc_ pattern in sleep-deprived animals resembled the pattern in these same regions observed under normal alert conditions (see Figure 8) suggests that the action of CX717 was to revert the brain to a state sufficient to perform the task successfully.

The functional linkage between brain regions engaged by the DMS task and their susceptibility to sleep deprivation and CX717 was demonstrated by the multivariate CDA analysis (Figure 9), which showed that brain metabolic changes (see Figures 7F–7H and 8A–8C) and behavioral performance (see Figures 2 and 4) in each test condition covaried within individual animals [17]. Three independent sources of variance were extracted from the covariance matrix, and each was determined to result from a major manipulation in the study; DMS task parameters (DF1), presence of CX717 (DF2), and exposure to 30–36 h of sleep deprivation (DF3). The CDA revealed that DF2 and DF3 were additive within individual animals and present when performance (DF1) was reversed in the sleep deprivation + CX717 condition (Figure 9A). This confirmed that the differential alterations in CMR_glc_ in specific brain regions by sleep deprivation (DPFC, DStr, and MTL; Figure 8) were specific to CX717 action in sleep-deprived animals (Figure 9B), suggesting that these areas were linked with respect to susceptibility to sleep deprivation and CX717 effects [17,44].

Although the finding that separate brain regions (DPFC, DStr, and MTL) were differentially altered by sleep deprivation (see Figure 8) is consistent with several human studies [15,16,45], recent reports indicate that generalization of such deficits to other circumstances and tasks is not straightforward [18,19,43,44]. The demands of sleep deprivation most likely engage different processes depending on both the type and the difficulty of the task [17,40,44]. It has been suggested that (1) different brain “circuits” may be activated under sleep deprivation conditions that are not engaged under normal conditions when performing the same tasks [17,44], and (2) that such demands are expressed differently depending on the specific regions that are “recruited” to perform the task [40,45]. At this time it is not possible to know whether such factors are similarly expressed with respect to sleep deprivation in nonhuman primates. The differential nature of the disruption in CMR_glc_ across different brain regions shown here, however, suggests that compensatory processes may have been involved.

The one brain area in which the effects of sleep deprivation were not ameliorated by CX717 was the thalamus, which showed very different CMR_glc_ patterns in all conditions compared to DPFC, DStr, precuneate cortex, and MTL (Figure 9B). While CMR_glc_ in the thalamus has been reported to be affected in sleep deprivation [17,44] other reports using functional magnetic resonance imaging actually found increased thalamic activation in sleep-deprived humans [15,40]. The lack of responsiveness to CX717 under either normal or sleep deprivation conditions in the present study (Figure 9B), however, provides new information regarding mechanisms of sleep regulation by the thalamus. The thalamic reticular nucleus, a major synchronizing influence on cortical EEG, contains clusters of electrically coupled neurons [46], which may not be directly affected by an AMPA receptor modulator such as CX717. This lack of direct synaptic influence could explain the sustained decrease in thalamic CMR_glc_ in the sleep deprivation + CX717 condition, and perhaps also why the associated behavioral performance (see Figure 4B) did not reverse to levels exhibited in the normal + CX717 condition (see Figure 2B).

Several investigations have shown that sleep deprivation down-regulates glutamatergic as well as other synaptic and receptor-mediated events in cortical neurons [47–50]. Ampakines modify ionic current through glutamate-gated channels with differential effectiveness across AMPA receptor subtypes, primarily by modulating desensitization and slowing channel closing [3–5,9,51]. The involvement in sleep and wakefulness of glutamatergic synapses along with several other neurotransmitter systems has been addressed in several studies [52]. AMPA receptor stimulation in the nucleus basalis of Meynert has been implicated in the activation of inhibitory projections from this nucleus to other brain regions as the basis for decreasing a particular type of sleep pattern [53]. Functional AMPA receptors have been shown in most of the affected brain regions described here [14]; hence, their enhancement by an agent such as CX717 could compensate for down-regulated glutamatergic systems in sleep-deprived subjects [50,54]. Another important neurotransmitter involved in sleep-waking cycles is orexin, a peptide that prevents sleep in narcoleptic dogs and has also been shown to release glutamate [52]. Consequently, the sleep-preventing effects of orexin may be potentiated by CX717. Ampakines, therefore, have the capability to positively modulate many different glutamatergic circuits and pathways in several pertinent brain regions [11], thereby increasing the potential to counteract cognitive deficits under normal as well as sleep deprivation conditions [16,55–57].

Other compounds that have been utilized to combat the effects of sleep deprivation, including psychostimulants (amphetamine), caffeine, and modafinil [58–62] act through different, non-AMPA receptor-mediated, cell signaling pathways. The usefulness of these agents, however, may be limited due to their potential for addiction and/or their potent stimulant actions, which can distort cognitive and sensory processes at doses required to counteract the effects of sleep deprivation [58–62].

In this investigation, the ampakine CX717 improved performance under both normal and sleep deprivation conditions in a nonhuman primate model. The effects on performance were accompanied by significant changes in local glucose metabolism in brain regions implicated in this type of cognitive processing. Selective activation of these brain regions over others during performance of the task, and reciprocal modulation by CX717 versus sleep deprivation, are key supportive outcomes of this study. The fact that ampakines such as CX717 can temporarily alleviate the effects of prolonged periods of sleep deprivation, even to the extent of improving performance above normal levels, indicates their potential applicability to many circumstances in which human performance is compromised by extensive sleep loss.

## Materials and Methods

### Subjects

All procedures were reviewed and approved by the Institutional Animal Care and Use Committee of Wake Forest University, and performed in accordance with established practices as described in the National Institutes of Health Guide for Care and Use of Laboratory Animals. In this study, 11 adult male rhesus monkeys *(Macaca mulatta)* weighing (8.0–11.0 kg) were utilized. They were individually housed in stainless steel cages in temperature- and humidity-controlled colony rooms with lighting maintained on a 0600:1800 on:off schedule, and were fed a diet of monkey chow supplemented by fresh fruit to maintain daily-monitored body weight. Fluid intake was restricted in time and amount such that a large volume of an animal's required daily fluid intake (80 ml/kg) was received either during the behavioral testing session, or within 2 h of being returned to the home cage.

### Behavioral testing

The number of sessions analyzed per condition (normal vehicle, normal + CX717, sleep deprivation, sleep deprivation + CX717) was 11–16, with nine monkeys tested at least once per condition, and two additional monkeys tested only under normal vehicle and normal + CX717 conditions. Animals were placed in a primate chair 1.5 m in front of an LCD-front-projection screen for daily testing on a visual DMS task [24] and performed 150–300 trials per session. Animals were trained to move a cursor tracked by a fluorescent marker attached to the back of the monkey's hand into the images by positioning the hand within a two-dimensional coordinate system referenced to an extended armrest on the chair. Stimuli consisted of clip art images projected as 25-cm squares within a 3 × 3 position matrix onto a 1.0 m × 1.0 m display. Responses to appropriate stimuli were rewarded with 0.5 ml of diluted fruit juice. Restricting most fluid intake to the task or 2 h after task completion ensured that animals were motivated to work for the liquid reinforcement. All animals were trained to a stable baseline on the DMS task in which delay varied randomly from 1 s to 30 s on a given trial, and the number of nonmatch stimuli (images) varied randomly from two to six in the match phase of the task (see Figure 1). Performance accuracy varied directly with duration of delay and number of nonmatch images (number of images) presented in the match phase. An individual image was used on only one trial per day, and the sets of images were routinely changed to maintain the trial-unique feature of each session.

### Sleep deprivation procedure

Sleep deprivation consisted of 30–36 h of continuous sleep prevention supervised by laboratory personnel. Animals were maintained in a cage separate from their home cages in a continuously lighted room and kept awake with videos, music, occasional treats, gentle cage shaking, and interaction with technicians until their usual daily testing time. EEG recordings were obtained in four animals from a set of five recording electrodes consisting of 1 mm diameter Ag/AgCl pellets on silver wire placed on the exposed dura around the circumference of a 1.5 cm diameter craniotomy access cylinder positioned over the parietal cortex and permanently attached to the skull. EEG was digitized at 200 Hz, and low pass filtered to 0.1–45 Hz. EEG was recorded during normal vehicle, sleep deprivation, and sleep deprivation + CX717 sessions, for 10 min before testing, and throughout the DMS testing session. Mean PSD was calculated from Fourier transforms of the EEG over different time epochs. Determination of sleepiness was assessed by the relative power of the EEG in terms of frequency bands: delta (0.5–5 Hz), alpha (8–12 Hz), sigma (spindles, 12–15 Hz), and beta (15–25 Hz). Transitions in EEG frequency during testing sessions were assessed by calculating PSDs in 4.0-s epochs over the entire session [25,26], which allowed insight into the basis of individual peaks in averaged PSD plots over longer time intervals (see Figure 6). Sleep deprivation sessions were conducted a minimum of 10 d apart with normal alert sessions interleaved to allow full recovery of baseline performance levels (typically within 24–48 h) following the sleep deprivation session.

### Drug administration

The ampakine CX717 (Cortex Pharmaceuticals, Irvine, California, United States) was administered in 10% w/v hydroxypropyl-beta-cyclodextrin and saline (0.45%) vehicle through an intravenous vascular access port chronically implanted in each monkey. CX717 was mixed as 1.5 mg/ml and administered within a dose range of 0.3–1.5 mg/kg to each monkey (weight range 8–11 kg). Vehicle injections (cyclodextrin and saline) were administered to all monkeys prior to nondrug sessions. CX717 in vehicle was administered 10 min prior to the start of each drug session. Normal sessions in which CX717 was administered were interposed between days with vehicle (normal vehicle) sessions. Recovery of baseline DMS performance was required prior to administration of CX717 in either sleep deprivation or normal vehicle sessions. In sessions in which PET scans were conducted, CX717 was administered 10 min prior to isotope injection.

### Measurement of local rates of cerebral glucose metabolism

Measurements of rates of cerebral glucose metabolism (CMR_glc_) were made in nine of the 11 monkeys in five different conditions: (1) baseline “no task” condition during which monkeys watched a video, (2) DMS task/normal vehicle, (3) DMS task/normal + CX717, (4) DMS task/sleep deprivation, and (5) DMS task/sleep deprivation + CX717. Animals were scanned twice in the baseline, normal vehicle, and normal + CX717 conditions. All animals (*n* = 9) were acclimated to the PET scan procedures, including blood collection, during several sessions prior to the initial scans in order to prevent disruption of performance. On the day of the scan the monkeys were placed in primate chairs and the DMS task initiated. Animals worked for ten trials prior to receiving a 30 s injection of 3–5 mCi of [^18^F]-2-deoxy-2-fluoro-D-glucose (FDG), after which they performed the DMS task for a total of 40 min. Animals were fully awake and behaving at all times during tracer uptake. Since the metabolized glucose tracer is taken up and trapped in cells for a minimum of 90 min after incorporation, measurement of the metabolized tracer reflected activation of brain regions during 80–100 trials of behavioral performance in the task. Following task completion, animals were anesthetized with ketamine (15 mg/kg IV) and transported to the PET scanner. Anesthesia was maintained by supplemental ketamine (10 mg/kg intramuscularly). Following scan acquisition, monkeys were transported back to their home cages and continuously monitored until fully recovered.

A total of three blood samples were collected from the femoral vein (opposite the catheter): 10 min prior to tracer injection, and 8 min and 42 min postinjection. Tracer concentrations were measured in blood samples using an automated gamma well counter. A population-averaged FDG blood curve (calculated for monkey) was scaled to the measured blood curve for the time period from injection to the end of the PET scan [63]. Data were transformed to CMR_glc_ based on the operational equation [64,65]. Rate constants (*k_1_, k_2_,* and *k_3_*) and lumped constant as determined in monkey by Kennedy et. al. [66], along with a *k_4_* value used in human studies [65] were applied.

Scan data were acquired with a General Electric (GE Medical Systems, Milwaukee, Wisconsin, United States) Advance NXi PET scanner with a resolution of 4 mm. Scanning consisted of a 5-min transmission scan acquired in 2D mode, followed by a 10-min emission scan acquired in 3D mode. The image reconstruction of the 3D data used the 3D-reprojection method with full quantitative corrections. Transmission scan data were smoothed using a 4-mm Gaussian filter transaxially and then segmented. The emission data were corrected for attenuation and reconstructed into 128 × 128 matrices using a Hanning filter with a 4-mm cutoff transaxially, and a ramp filter with an 8.5-mm cutoff axially.

PET data were analyzed with Statistical Parametric Mapping (SPM99) software (University College London, London, United Kingdom; http://www.fil.ion.ucl.ac.uk/spm/) implemented in MATLAB (MathWorks, Natick, Massachusetts, United States). Reconstructed images for each scan from each of the nine monkeys were co-registered to corresponding structural MR images obtained on a 1.5-T MR scanner (GE Medical Systems) using automated image registration [67] and then transformed spatially into a standard space with an FDG template for rhesus monkeys constructed in our laboratory based on procedures of Black and colleagues [68]. Resultant images were smoothed using a 2 mm isotropic Gaussian kernel with a voxel size of 1 mm × 1 mm × 1 mm. Scans were normalized for differences in global activity by proportional scaling.

Effects at each voxel were estimated according to the general linear model using the multi-subject conditions and covariates option in SPM99 software. Statistical parametric maps were created for the following comparisons: normal vehicle versus baseline (no task); normal + CX717 versus normal vehicle; sleep deprivation versus normal vehicle; and sleep deprivation versus sleep deprivation + CX717. Exploratory analyses used a minimum voxel height (magnitude) threshold of *p* < 0.01 and a minimum cluster size of 50 voxels. A region of interest analysis was conducted including DPFC, MTL, parietal cortex (precuneus), thalamus, and DStr, selected based on previous imaging reports in nonhuman primates implicating these regions in delay and working memory tasks [37,38]. Spherical ROIs were constructed on a structural MR template using the MarsBaR toolbox contained in SPM99. Statistical significance for the four comparisons described above was determined with a threshold value of *p* < 0.05 corrected for the search volume. Areas of activation were displayed on a T_1_ weighted anatomic MRI template [68]. Regions were then identified with reference to standard atlases of the primate brain [69].

Further analyses were conducted on the PET data for use with multivariate analyses. Each of the PET datasets as described above was co-registered to structural MR images, and a template of ROIs created on the MR image. ROIs were chosen on the basis of the results of the SPM analysis and included the same five brain areas listed above: DPFC, parietal cortex, DStr, thalamus, MTL, plus cingulate cortex. Absolute rates of CMR_glc_ were determined bilaterally for each animal for each condition. These data were used for the multivariate canonical discriminant analysis.

### Multivariate analysis of performance and brain glucose utilization

To conclusively demonstrate that measures of DMS performance and CMR_glc_ covaried within animals across the four test conditions (normal vehicle, normal + CX717, sleep deprivation, and sleep deprivation + CX717), a multivariate CDA [70] was performed. The CDA extracted common sources of variance from a covariance matrix that included all relevant dimensions of the study [32], including measures of behavioral performance and CMR_glc_ from individual sessions in nine different monkeys. There were six behavioral measures: (1) percent correct over all trials, (2) percent correct on the most difficult trials (21–30 s delays and 5–6 images), (3) percent correct on the least difficult trials (1–10 s delays and 2–3 images), (4) response latency to start the trial, (5) latency to sample response, and (6) latency to match response. The other six measures consisted of absolute levels of CMR_glc_ from six different brain regions: (7) DPFC, (8) DStr, (9) MTL, (10) thalamus, (11) precuneus, and (12) cingulate cortex, determined by separate ROIs computed specifically for the CDA. All 12 of the above measures were determined for each of nine monkeys under the four different test conditions.

The CDA program (PROC CANDISC, SAS Institute, Cary, North Carolina, United States) constructed canonical covariance matrices between dependent variables (behavioral and CMR_glc_ measures) across each of the four conditions, then performed an eigenvector decomposition (“factoring”) to produce normalized, orthogonal DFs such that:



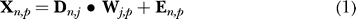



Where *n* = number of observations, *p* = number of dependent variables, *j* = number of eigenvectors (extracted by the CDA), **X** is the matrix of raw data, **D** is the matrix of discriminant scores, **W** is the matrix of eigenvectors (i.e., coefficients of the DFs), **E** is the matrix of residual error, and • indicates the matrix dot-product. DF scores for each monkey and condition were computed from the coefficients via the formula:



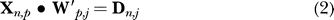



Where **X** comprises the raw data matrix, with *n,* *p,* and *j* indices as described above, **W**′ is the inverse of the eigenvector coefficient matrix, and **D** is the matrix of discriminant scores. The eigenvectors were orthogonally rotated to (1) distribute scores around a mean of zero, with standard deviation of one, and (2) maximize the Mahalonobis distances between measures within treatment conditions. With *n* = 36 (9 monkeys × 4 conditions), *p* = 12 (dependent variables), and *j* = 3 (DFs), 108 discriminant scores were produced and tested by two-way ANOVA for differences between the four test conditions. Variance contribution for each DF from the matrix of eigenvalues (**V**, produced concomitantly with the eigenvector decomposition) was compared with the sum *(v)* of all eigenvalues and residual error (**E**, above) to derive the proportion of total variance accounted for by each DF [70].

## Abbreviations

AMPA: α-amino-3-hydroxy-5-methyl-4-isoxazolepropionic acid
CDA: canonical discriminant analysis
CMR_glc_: cerebral metabolic rate for glucose
DF: discriminant function
DMS: delayed match-to-sample
DPFC: dorsal prefrontal cortex
DStr: dorsal striatum
EEG: electroencephalogram
IV: intravenous
MTL: medial temporal lobe
NS: not significant
PET: positron emission tomography
PSD: power spectral density
ROI: region of interest
SEM: standard error of the mean
SPM: statistical parametric map

## References

[pbio-0030299-b01] Schmitt WB, Sprengel R, Mack V, Draft RW, Seeburg PH (2005). Restoration of spatial working memory by genetic rescue of GluR-A-deficient mice. Nat Neurosci.

[pbio-0030299-b02] Rumpel S, Ledoux J, Zador A, Malinow R (2005). Postsynaptic receptor trafficking underlying a form of associative learning. Science.

[pbio-0030299-b03] Suppiramaniam V, Bahr BA, Sinnarajah S, Owens K, Rogers G (2001). Member of the Ampakine class of memory enhancers prolongs the single channel open time of reconstituted AMPA receptors. Synapse.

[pbio-0030299-b04] Arai AC, Kessler M, Rogers G, Lynch G (2000). Effects of the potent ampakine CX614 on hippocampal and recombinant AMPA receptors: Interactions with cyclothiazide and GYKI 52466. Mol Pharmacol.

[pbio-0030299-b05] Arai AC, Xia YF, Rogers G, Lynch G, Kessler M (2002). Benzamide-type AMPA receptor modulators form two subfamilies with distinct modes of action. J Pharmacol Exp Ther.

[pbio-0030299-b06] Larson J, Lieu T, Petchpradub V, LeDuc B, Ngo H (1995). Facilitation of olfactory learning by a modulator of AMPA receptors. J Neurosci.

[pbio-0030299-b07] Granger R, Deadwyler SA, Davis M, Moskovitz B, Kessler M (1996). Facilitation of glutamate receptors reverses an age-associated memory impairment in rats. Synapse.

[pbio-0030299-b08] Hampson RE, Rogers G, Lynch G, Deadwyler SA (1998). Facilitative effects of the ampakine CX516 on short-term memory in rats: Correlations with hippocampal ensemble activity. J Neurosci.

[pbio-0030299-b09] Buccafusco JJ, Weiser T, Winter K, Klinder K, Terry AV (2004). The effects of IDRA 21, a positive modulator of the AMPA receptor, on delayed matching performance by young and aged rhesus monkeys. Neuropharmacology.

[pbio-0030299-b10] Goff DC, Leahy L, Berman I, Posever T, Herz L (2001). A placebo-controlled pilot study of the ampakine CX516 added to clozapine in schizophrenia. J Clin Psychopharmacol.

[pbio-0030299-b11] Lynch G (2002). Memory enhancement: The search for mechanism-based drugs. Nat Neurosci.

[pbio-0030299-b12] Ingvar M, Ambros-Ingerson J, Davis M, Granger R, Kessler M (1997). Enhancement by an ampakine of memory encoding in humans. Exp Neurol.

[pbio-0030299-b13] Turrigiano GG, Nelson SB (1998). Thinking globally, acting locally: AMPA receptor turnover and synaptic strength. Neuron.

[pbio-0030299-b14] Takahashi T, Svoboda K, Malinow R (2003). Experience strengthening transmission by driving AMPA receptors into synapses. Science.

[pbio-0030299-b15] Chee MW, Choo WC (2004). Functional imaging of working memory after 24 hr of total sleep deprivation. J Neurosci.

[pbio-0030299-b16] Drummond SP, Brown GG (2001). The effects of total sleep deprivation on cerebral responses to cognitive performance. Neuropsychopharmacology.

[pbio-0030299-b17] Habeck C, Rakitin BC, Moeller J, Scarmeas N, Zarahn E (2004). An event-related fMRI study of the neurobehavioral impact of sleep deprivation on performance of a delayed-match-to-sample task. Brain Res Cogn Brain Res.

[pbio-0030299-b18] Choo WC, Lee WW, Venkatraman V, Sheu FS, Chee MW (2005). Dissociation of cortical regions modulated by both working memory load and sleep deprivation and by sleep deprivation alone. Neuroimage.

[pbio-0030299-b19] Drummond SP, Gillin JC, Brown GG (2001). Increased cerebral response during a divided attention task following sleep deprivation. J Sleep Res.

[pbio-0030299-b20] Edell-Gustaffson UM (2002). Insufficient sleep, cognitive anxiety and health transition in men with coronary artery disease: A self-report and polysomnographic study. J Adv Nurs.

[pbio-0030299-b21] Horne J, Reyner L (1999). Vehicle accidents related to sleep: A review. Occup Environ Med.

[pbio-0030299-b22] Caldwell JL, Prazinko BF, Rowe T, Norman D, Hall KK (2003). Improving daytime sleep with temazepam as a countermeasure for shift lag. Aviat Space Environ Med.

[pbio-0030299-b23] Nilsson JP, Soderstrom M, Karlsson AU, Lekander M, Akerstedt T (2005). Less effective executive functioning after one night's sleep deprivation. J Sleep Res.

[pbio-0030299-b24] Hampson RE, Pons TP, Stanford TR, Deadwyler SA (2004). Categorization in the monkey hippocampus: A possible mechanism for encoding information into memory. Proc Natl Acad Sci U S A.

[pbio-0030299-b25] Benca RM, Obermeyer WH, Larson CL, Yun B, Dolski I (1999). EEG alpha power and alpha power asymmetry in sleep and wakefulness. Psychophysiology.

[pbio-0030299-b26] Benca RM, Obermeyer WH, Shelton SE, Droster J, Kalin NH (2000). Effects of amygdala lesions on sleep in rhesus monkeys. Brain Res.

[pbio-0030299-b27] Anderson C, Horne JA (2003). Prefrontal cortex: Links between low frequency delta EEG in sleep and neuropsychological performance in healthy, older people. Psychophysiology.

[pbio-0030299-b28] Merica H, Fortune RD (2005). Spectral power time-courses of human sleep EEG reveal a striking discontinuity at ∼18 Hz marking the division between NREM-specific and wake/REM-specific fast frequency activity. Cereb Cortex.

[pbio-0030299-b29] De Carli F, Nobili L, Beelke M, Watanabe T, Smerieri A (2004). Quantitative analysis of sleep EEG microstructure in the time-frequency domain. Brain Res Bull.

[pbio-0030299-b30] Hemmeter U, Bischof R, Hatzinger M, Seifritz E, Holsboer-Trachsler E (1998). Microsleep during partial sleep deprivation in depression. Biol Psychiatry.

[pbio-0030299-b31] Landolt HP, Retey JV, Tonz K, Gottselig JM, Khatami R (2004). Caffeine attenuates waking and sleep electroencephalographic markers of sleep homeostasis in humans. Neuropsychopharmacology.

[pbio-0030299-b32] Deadwyler SA, Hampson RE (1997). The significance of neural ensemble codes during behavior and cognition. Annu Rev Neurosci.

[pbio-0030299-b33] Cook EP, Maunsell JH (2004). Attentional modulation of motion integration of individual neurons in the middle temporal visual area. J Neurosci.

[pbio-0030299-b34] Malkova L, Mishkin M (2003). One-trial memory for object-place associations after separate lesions of hippocampus and posterior parahippocampal region in the monkey. J Neurosci.

[pbio-0030299-b35] Manns JR, Hopkins RO, Reed JM, Kitchener EG, Squire LR (2003). Recognition memory and the human hippocampus. Neuron.

[pbio-0030299-b36] Freedman DJ, Riesenhuber M, Poggio T, Miller EK (2003). A comparison of primate prefrontal and inferior temporal cortices during visual categorization. J Neurosci.

[pbio-0030299-b37] Friedman HR, Goldman-Rakic PS (1994). Coactivation of prefrontal cortex and inferior parietal cortex in working memory tasks revealed by 2DG functional mapping in the rhesus monkey. J Neurosci.

[pbio-0030299-b38] Inoue M, Mikami A, Ando I, Tsukada H (2004). Functional brain mapping of the macaque related to spatial working memory as revealed by PET. Cereb Cortex.

[pbio-0030299-b39] Daselaar SM, Veltman DJ, Witter MP (2004). Common pathway in the medial temporal lobe for storage and recovery of words as revealed by event-related functional MRI. Hippocampus.

[pbio-0030299-b40] Drummond SP, Brown GG, Salamat JS, Gillin JC (2004). Increasing task difficulty facilitates the cerebral compensatory response to total sleep deprivation. Sleep.

[pbio-0030299-b41] Drummond SP, Brown GG, Gillin JC, Stricker JL, Wong EC (2000). Altered brain response to verbal learning following sleep deprivation. Nature.

[pbio-0030299-b42] Harrison Y, Horne JA, Rothwell A (2000). Prefrontal neuropsychological effects of sleep deprivation in young adults—A model for healthy aging?. Sleep.

[pbio-0030299-b43] Rogers NL, Dorrian J, Dinges DF (2003). Sleep, waking and neurobehavioural performance. Front Biosci.

[pbio-0030299-b44] Bell-McGinty S, Habeck C, Hilton HJ, Rakitin B, Scarmeas N (2004). Identification and differential vulnerability of a neural network in sleep deprivation. Cereb Cortex.

[pbio-0030299-b45] Drummond SP, Smith MT, Orff HJ, Chengazi V, Perlis ML (2004). Functional imaging of the sleeping brain: Review of findings and implications for the study of insomnia. Sleep Med Rev.

[pbio-0030299-b46] Long MA, Landisman CE, Connors BW (2004). Small clusters of electrically coupled neurons generate synchronous rhythms in the thalamic reticular nucleus. J Neurosci.

[pbio-0030299-b47] Pace-Schott EF, Hobson JA (2002). The neurobiology of sleep: Genetics, cellular physiology and subcortical networks. Nat Rev Neurosci.

[pbio-0030299-b48] Siegel JM (2001). The REM sleep-memory consolidation hypothesis. Science.

[pbio-0030299-b49] Stickgold R, Hobson JA, Fosse R, Fosse M (2001). Sleep, learning, and dreams: Off-line memory reprocessing. Science.

[pbio-0030299-b50] Graves L, Pack A, Abel T (2001). Sleep and memory: A molecular perspective. Trends Neurosci.

[pbio-0030299-b51] Quirk JC, Nisenbaum ES (2002). LY404187: A novel positive allosteric modulator of AMPA receptors. CNS Drug Rev.

[pbio-0030299-b52] Siegel JM (2004). The neurotransmitters of sleep. J Clin Psychiatry.

[pbio-0030299-b53] Manfridi A, Brambilla D, Mancia M (2001). Sleep is differently modulated by basal forebrain GABA(A) and GABA(B) receptors. Am J Physiol Regul Integr Comp Physiol.

[pbio-0030299-b54] McDermott CM, LaHoste GJ, Chen C, Musto A, Bazan NG (2003). Sleep deprivation causes behavioral, synaptic, and membrane excitability alterations in hippocampal neurons. J Neurosci.

[pbio-0030299-b55] Smith ME, McEvoy LK, Gevins A (2002). The impact of moderate sleep loss on neurophysiologic signals during working-memory task performance. Sleep.

[pbio-0030299-b56] Thomas M, Sing H, Belenky G, Holcomb H, Mayberg H (2000). Neural basis of alertness and cognitive performance impairments during sleepiness. I. Effects of 24 h of sleep deprivation on waking human regional brain activity. J Sleep Res.

[pbio-0030299-b57] Ferrara M, De Gennaro L, Casagrande M, Bertini M (2000). Selective slow-wave sleep deprivation and time-of-night effects on cognitive performance upon awakening. Psychophysiology.

[pbio-0030299-b58] Beaumont M, Batejat D, Pierard C, Coste O, Doireau P (2001). Slow release caffeine and prolonged (64-h) continuous wakefulness: Effects on vigilance and cognitive performance. J Sleep Res.

[pbio-0030299-b59] Wiegmann DA, Stanny RR, McKay DL, Neri DF, McCardie AH (1996). Methamphetamine effects on cognitive processing during extended wakefulness. Int J Aviat Psychol.

[pbio-0030299-b60] Wesensten NJ, Belenky G, Kautz MA, Thorne DR, Reichardt RM (2002). Maintaining alertness and performance during sleep deprivation: Modafinil versus caffeine. Psychopharmacology (Berl).

[pbio-0030299-b61] Martinez-Gonzalez D, Obermeyer W, Fahy JL, Riboh M, Kalin NH (2004). REM sleep deprivation induces changes in coping responses that are not reversed by amphetamine. Sleep.

[pbio-0030299-b62] Wesensten NJ, Belenky G, Thorne DR, Kautz MA, Balkin TJ (2004). Modafinil vs. caffeine: Effects on fatigue during sleep deprivation. Aviat Space Environ Med.

[pbio-0030299-b63] Takikawa S, Dhawan V, Spetsieris P, Robeson W, Chaly T (1993). Noninvasive quantitative fluorodeoxyglucose PET studies with an estimated input function derived from a population-based arterial blood curve. Radiology.

[pbio-0030299-b64] Sokoloff L, Reivich M, Kennedy C, Des Rosiers MH, Patlak CS (1977). The [^14^C]deoxyglucose method for the measurement of local cerebral glucose utilization: Theory, procedure, and normal values in the conscious and anesthetized albino rat. J Neurochem.

[pbio-0030299-b65] Phelps ME, Huang SC, Hoffman EJ, Selin C, Sokoloff L (1979). Tomographic measurement of local cerebral glucose metabolic rate in humans with (F-18)2-fluoro-2-deoxy-D-glucose: Validation of method. Ann Neurol.

[pbio-0030299-b66] Kennedy C, Sakurada O, Shinohara M, Jehle J, Sokoloff L (1978). Local cerebral glucose utilization in the normal conscious macaque monkey. Ann Neurol.

[pbio-0030299-b67] Woods RP, Mazziotta JC, Cherry SR (1993). MRI-PET registration with automated algorithm. J Comput Assist Tomogr.

[pbio-0030299-b68] Black KJ, Koller JM, Snyder AZ, Perlmutter JS (2001). Template images for nonhuman primate neuroimaging: 2. Macaque. Neuroimage.

[pbio-0030299-b69] Paxinos G, Huang XF, Toga AW (2003). The Rhesus monkey brain in stereotaxic coordinates.

[pbio-0030299-b70] Stevens J (1992). Applied multivariate statistics for the social sciences.

